# LL-37—biofunctionalized titanium improves soft tissue seal surrounding the dental implant from the perspective of optimizing a race to the surface

**DOI:** 10.1093/rb/rbaf117

**Published:** 2025-11-12

**Authors:** Yi Li, Junling Huang, Yan Zhang, Yide He, Dongxuan Cai, Min Xu, Qianli Ma, Yumei Zhang, Jinjin Wang

**Affiliations:** State Key Laboratory of Military Stomatology & National Clinical Research Center for Oral Diseases & Shaanxi Key Laboratory of Oral Diseases, Department of Prosthodontics, School of Stomatology, The Fourth Military Medical University, Xi’an 710032, China; State Key Laboratory of Oral & Maxillofacial Reconstruction and Regeneration, National Clinical Research Center for Oral Diseases, Shaanxi International Joint Research Center for Oral Diseases, Department of Periodontology, School of Stomatology, The Fourth Military Medical University, Xi’an 710032, China; State Key Laboratory of Military Stomatology & National Clinical Research Center for Oral Diseases & Shaanxi Key Laboratory of Oral Diseases, Department of Prosthodontics, School of Stomatology, The Fourth Military Medical University, Xi’an 710032, China; State Key Laboratory of Military Stomatology & National Clinical Research Center for Oral Diseases & Shaanxi Key Laboratory of Oral Diseases, Department of Prosthodontics, School of Stomatology, The Fourth Military Medical University, Xi’an 710032, China; State Key Laboratory of Military Stomatology & National Clinical Research Center for Oral Diseases & Shaanxi Key Laboratory of Oral Diseases, Department of Prosthodontics, School of Stomatology, The Fourth Military Medical University, Xi’an 710032, China; State Key Laboratory of Military Stomatology & National Clinical Research Center for Oral Diseases & Shaanxi Key Laboratory of Oral Diseases, Department of Prosthodontics, School of Stomatology, The Fourth Military Medical University, Xi’an 710032, China; Department of Biomaterials, Institute of Clinical Dentistry, University of Oslo, Oslo 0445, Norway; State Key Laboratory of Military Stomatology & National Clinical Research Center for Oral Diseases & Shaanxi Key Laboratory of Oral Diseases, Department of Prosthodontics, School of Stomatology, The Fourth Military Medical University, Xi’an 710032, China; State Key Laboratory of Oral & Maxillofacial Reconstruction and Regeneration, National Clinical Research Center for Oral Diseases, Shaanxi International Joint Research Center for Oral Diseases, Department of Periodontology, School of Stomatology, The Fourth Military Medical University, Xi’an 710032, China

**Keywords:** LL-37, soft tissue seal, dental implant, human gingival fibroblasts, antibacterial

## Abstract

The bacterial oral environment poses a significant challenge to the long-term stability of dental implants due to vulnerability of peri-implant soft tissues to pathogenic infiltration. Therefore, the rapid formation of a dense soft tissue barrier in the transgingival mucosal area surrounding the implant is essential. In this study, we engineer a biofunctionalized titanium (Ti) material by leveraging polydopamine (PD) as an intermediate coating to immobilize the peptide LL-37 onto nanostructured Ti substrates (LL-37-PD@NT). Material characterization shows that LL-37 is successfully loaded onto Ti substrate, and although the roughness of LL-37-PD@NT increases within a certain extent, the overall biological activity is still better than that of smooth Ti, which is considered to be traditional abutment material; meanwhile, LL-37 can be released stably for more than 1 week. Furthermore, the *in vitro* experiments demonstrate dual functionality of LL-37-PD@NT: the modified Ti samples significantly promote the migration, adhesion, proliferation and ECM synthesis of human gingival fibroblasts (hGFs), while exhibiting potent antibacterial efficacy against *Pg* and *Sm*. In a rat model of implantation immediately after tooth extraction, a peri-implant epithelial structure resembling the junctional epithelium of natural teeth is observed surrounding dental implant of LL-37-PD@NT at 4 weeks, and the prevention for HRP penetration exhibits the potent sealing capacity of peri-implant soft tissues. Collectively, our findings validate that the LL-37—biofunctionalized Ti can simultaneously enhance hGFs’ biological functions and bacteriostatic performance, thus promoting formation and strength of soft tissue seal, holding promise as a novel option for implant abutment material.

## Graphical Abstract

**Figure:**
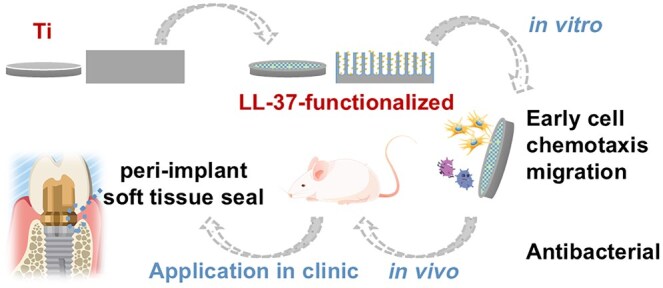

## Introduction

Once implanted, biomaterials typically face two major events: the adhesion of microbiota and the integration with host cells, which was described as ‘a race to the surface’ by Gristina, and the outcome of the above race was crucial for the prognosis of implant [1]. Dental implant, as a widely used type of biomaterial, is no exception. Especially the abutment that was located at the cervical region of dental implant, spanning through the gingival mucosa, leading to the susceptibility to bacterial invasion. Therefore, a good soft tissue seal between the abutment material and the gingiva is essential and serves as a critical barrier for osseointegration of the implant.

After implantation, the abutment material interacts with host cells positively, involving protein adsorption, immunocytes infiltration, such as neutrophils and monocytes/macrophages, and tissue cells adhesion, such as epithelial cells, fibroblasts and endothelial cells; consequently, epithelial attachment and connective tissue attachment are formed [2]. Among the tissue cells, human gingival fibroblasts (hGFs) are key components of the connective tissue attachment. However, compared to the natural teeth, the proportion of fibroblasts in peri-implant connective tissue attachment is only 3%, representing a significant reduction in quantity. Additionally, collagen fibers are parallel to the implant surface, lacking collagen fibers that are vertically embedded into the cementum [3]. Based on the above physiological reasons, the peri-implant connective tissue attachment might have poor sealing strength. Meanwhile, unlike osseointegration, the formation of peri-implant soft tissue seal occurs in the oral bacterial environment [4]. To sum up, the internal and external threats pose challenges to the rapid formation of excellent soft tissue seal, which should be the crucial precondition for the long-term retention of the implant. Therefore, how to achieve the rapid formation of excellent soft tissue seal had been a research hotspot. It is worth noting that endowing the abutment material with both favorable biocompatibility and antibacterial properties, thus helping the ‘surface race’ favor the prognosis of the implant is an important strategy.

Currently, various abutment materials are employed in clinical, such as pure titanium (Ti), zirconia and polyetheretherketone [5, 6]. Among these, pure Ti abutments remain the most widely used owing to their excellent mechanical strength, good biocompatibility and certain sterilization properties [7–9]. Research has always focused on developing the multifunctional Ti abutments that possess both excellent antimicrobial efficacy and enhanced biological activity [10–12]. However, the fabrication process was mostly quite complex, which almost involved two or more different functional bio-molecules loaded onto the material surface, and might result in high costs of extraction and purification and unclear interactions between multiple bio-molecules, which limited the clinical application. As a consequence, we have been committed to constructing a surface-modified Ti that is simple to prepare, inexpensive in costs, and possessing multiple biological activities, in order to meet the needs of implant abutment.

LL-37 is a multifunctional human antimicrobial peptide that participates in various physiological functions, such as cell recruitment and migration, immune regulation and bactericidal process, which made LL-37 might be an ideal choice for the modification of abutmen material [13–19]. The loading of LL-37 was expected to promote the recruitment and migration of hGFs, and exert antibacterial effects at the same time, ultimately promoting the ‘surface race’ favorable to tissue integration. To achieve highly efficient loading of LL-37, we selected the titanium dioxide (TiO_2_) nanotubes with a top-open and bottom-closed structure for drug storage, which have been successfully prepared by our research team [20]. Moreover, polydopamine (PD) was used as the intermediate coating for attaching LL-37 into the TiO_2_ nanotubes, considering its adhesive properties [21]. In consequence, we aimed to construct the LL-37 bio-functionally modified TiO_2_ nanotubes, and preferred to observe its *in vitro* effects on the chemotaxis, adhesion, proliferation, migration, cytokines secretion and collagen deposition of hGFs, and antibacterial action as well, then further verified its *in vivo* induction effects on peri-implant soft tissue seal, thus providing a new idea for optimal design of implant abutment material.

## Materials and methods

### Material preparation and characterization

#### Preparation of Ti samples

Pure Ti disks (10 mm in diameter, 1.5 mm in thickness) were provided by Baoji Titanium Industry Co. Ltd, Shaanxi, China. The disks were polished with SiC sandpaper from 400# to 7000# and were ultrasonically cleaned with acetone, ethanol and distilled water to generate the polished Ti samples (P). Subsequently, the nano-topography (NT) was fabricated by anodization in accordance with our previous reports [20]. For the anodization, the P samples were immersed into an electrolyte containing 372 mL of deionized water, 23 mL of 85% phosphate and 5 mL of hydrofluoric acid at a constant voltage of 20 V for 1 h.

To obtain the PD coating, the NT samples were immersed in 500 mL dopamine hydrochloride solution (2 mg/mL) with 10 mM Tris buffer (pH 8.5) and incubated 18 h. After that, the obtained samples were cleaned with distilled water and were labeled as PD-coated nanotubes (PD@NT).

**Figure 1.**
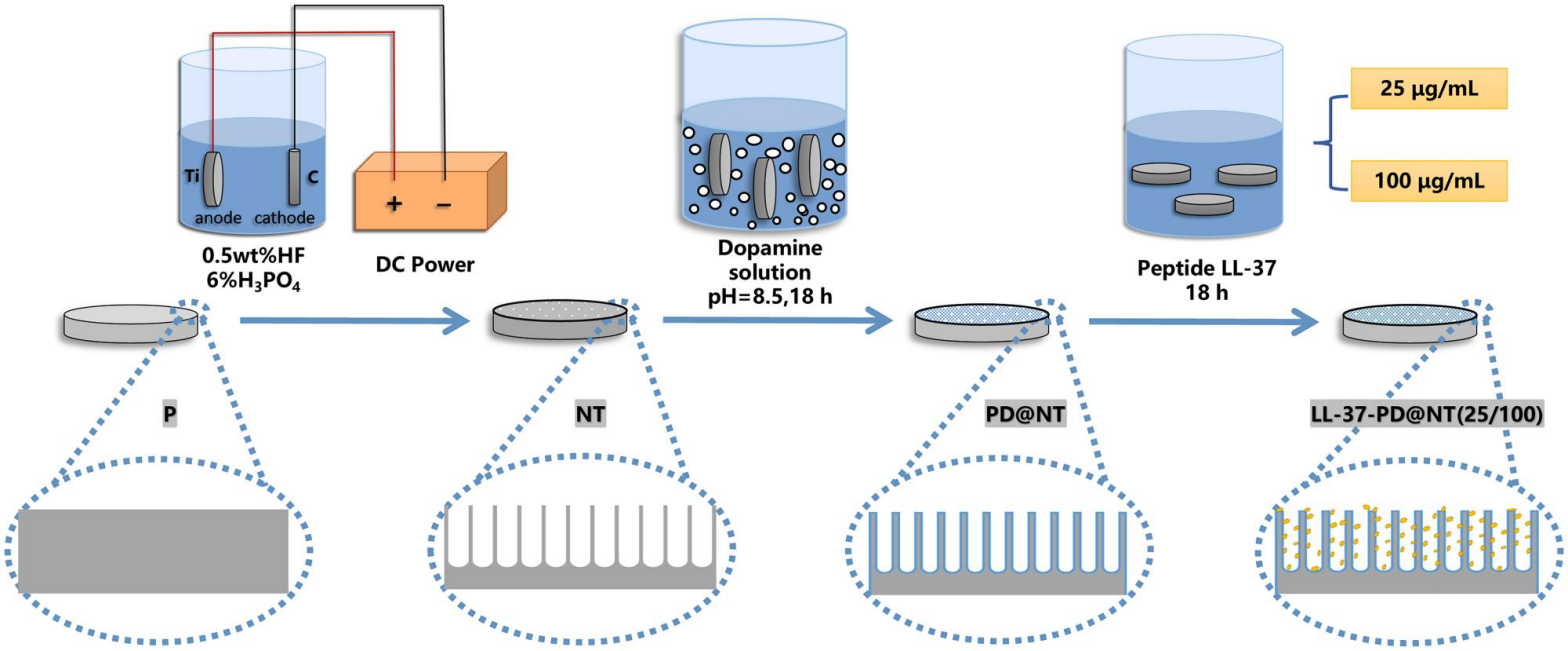
Preparation of Ti samples.

Subsequently, the samples were immersed in 10 mL LL-37 solutions with different concentrations at room temperature for 18 h. In order to determine the optimal loading concentration of LL-37, a preliminary experiment was conducted to investigate the influence of different LL-37 concentrations on the migration of hGFs. Detailed descriptions of preliminary experiment are provided in the Supplementary Section S1.1. The results demonstrated that 5 μg/mL of LL-37 exhibited the most pronounced promigratory effect (Supplementary Figure S1), which is similar to previous report [16, 22]. Additionally, guided by the loading concentrations reported in relevant literature, 25 and 100 μg/mL were ultimately selected as the low and high concentration groups, respectively [23]. The samples were denoted as LL-37-PD@NT (25) and LL-37-PD@NT (100). The sample preparation process is shown in Figure 1.

#### Characterization of Ti samples

The surface nanostructure of the prepared samples was observed using a field emission scanning electron microscope (FE-SEM, Hitachi, Japan) after drying, and the longitudinal view was acquired by cutting the samples at the midpoint. The surface roughness was determined by atomic force microscope (AFM, Shimadzu, Japan). The hydrophilicity was measured by the DSA1 System (Krüss, Hamburg, Germany). X-ray photoelectron spectroscopy (XPS, Thermo Fisher Scientific, America) was employed to characterize the chemical element components of different samples.

To explore the loading and releasing characteristics of peptide on LL-37-PD@NT, LL-37 was labeled with fluorescein isothiocyanate (FITC) [23]. FITC-labeled LL-37 was loaded onto the samples using the same immersion method as described in the section ‘Preparation of Ti samples’. Confocal laser scanning microscope (CLSM, Nikon, Japan) was used to identify the successful loading of LL-37. Then, to study the release profile of LL-37, the prepared LL-37-PD@NT (25) and LL-37-PD@NT (100) Ti samples were placed in 24-well plates with three replicates each and incubated with 1 mL phosphate-buffered saline (PBS) for each well in the dark. After 1, 2, 3, 4, 24, 48, 72, 96, 120, 170 and 220 h, 500 μL supernatant was collected and refreshed with new PBS solution. The fluorescence intensity of collected solutions was measured using a microplate absorbance reader and the cumulative release of FITC-labeled LL-37 was determined by converting the values according to the standard curve.

### Cell experiments

#### Cell culture

hGFs were purchased from American Type Culture Collection (Manassas, VA, USA) and were cultured in Dulbecco’s modified Eagle medium containing 10% fetal bovine serum (Gibco, Thermo Fischer Scientific, USA) and 1% penicillin/streptomycin. The cells were incubated in a humidified atmosphere of 5% CO_2_ at 37°C and the medium was changed every other day.

#### Cell chemotaxis and adhesion

**Figure 2.**
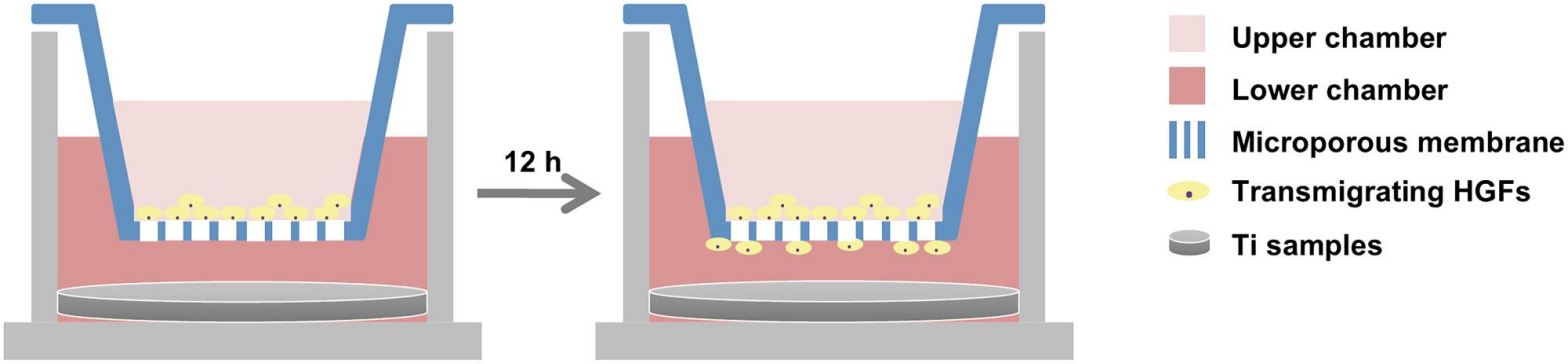
Schematic illustration of hGFs’ chemotactic experiments.

Transwell assay was used to investigate the chemotaxis of LL-37-PD@NT on hGFs (Figure 2). The different Ti samples (P, NT, PD@NT, LL-37-PD@NT (25) and LL-37-PD@NT (100)) were placed into 24-well plates, then the chambers (8 μm of pore size, Corning, USA) were placed above the Ti samples. hGFs were seeded onto the upper chamber with 5 × 10^4^ cells/well and medium was added to the lower chamber. After co-culturing for 12 and 24 h, a cotton swab was used to remove the remaining cells on the surface of upper chamber. The migrated cells on the bottom of upper chamber were fixed with 4% paraformaldehyde (PFA) and stained with 0.05% crystal violet. Images were recorded by a stereomicroscope and three visual fields were randomly selected from each group for counting.

To observe early cell adhesion, hGFs were seeded onto different Ti samples in 24-well plates at a density of 1 × 10^5^ cells per well. After 0.5 and 2 h, the culture medium was removed. The cells were then fixed with 4% PFA at room temperature for 30 min, followed by staining with Hoechst 33342 (Sigma, USA) for 10 min in the dark to mark the nucleus. CLSM was used to observe the adhesion of hGFs on different Ti samples, and the cells were measured and analyzed using ImageJ software.

#### Cell morphology and proliferation

hGFs were seeded onto different Ti samples in 24-well plates with 5 × 10^4^ cells/well. After 4 and 24 h, the culture medium was removed. The cells were fixed with 2.5% glutaraldehyde overnight and dehydrated with graded ethanol. After dried with hexamethyl disilylamine (Macklin, China), the Ti samples were sputter-coated with gold and observed by FE-SEM.

To observe cytoskeletal changes, the cells were fixed with 4% PFA at room temperature for 30 min. Then hGFs were incubated with Alexa Fluor 488 phalloidin (Invitrogen) for 40 min and Hoechst 33342 for 10 min in the dark to mark actin filaments and nucleus, respectively. CLSM was used to observe the morphology of hGFs on the surface of different Ti samples, and the length and length-width ratio of cells were measured and analyzed using ImageJ software.

3-(4, 5-Dimethylthiazol-2, 5-diphenyl tetrazolium bromide) (MTT) assay was applied to evaluate cell viability. hGFs were seeded onto different Ti samples in 24-well plates with 3 × 10^4^ cells/well. After culture for 1, 4 and 7 days, the culture medium was gently discarded and cells were washed with PBS twice, then MTT working solution was added into the well. After continuous incubation for another 4 h at 37°C with 5% CO_2_, the working solution was removed, and 500 μL dimethyl sulfoxide was added to dissolve the blue formazan crystals. The absorbance value of collected solutions from different samples was measured by microplate reader at 570 nm.

#### Cell migration

The wound scratch assay was performed to evaluate the cell migration ability of hGFs. To create the wound model, a 3-well culture-insert (ibidi, Germany) was placed onto the surface of each Ti sample, which had been positioned in the separate well of a 24-well plate. Then 70 µL cell suspension with the concentration of 3 × 10^5^ cells/mL was added into each well of the insert to obtain a confluent cell layer after 24 h at 37°C and 5% CO_2_. Then the culture-insert was removed and the culture medium containing 2% serum was added. After another 12 and 24 h, cells were rinsed with PBS thrice and fixed with 4% PFA for 30 min. Cellular immunofluorescence staining was used to observe the migration of hGFs and wound closure on different Ti samples at 0, 12 and 24 h, and the procedure of cell immunofluorescence staining is similar to the section ‘Cell morphology and proliferation’. The unhealing areas were quantified using the ImageJ wound-healing measurement tool. The healing rate was calculated by dividing the healing area by the total wound area.

#### Secretion of growth factors

The secretion of cell growth factors, including hepatocyte growth factor (HGF), basic fibroblast growth factor (bFGF) and keratinocyte growth factor (KGF) was detected using enzyme-linked immunosorbent assay (ELISA). Briefly, the supernatants of hGFs cultured on different Ti samples for 4 h and 7 days were collected, then the supernatants were centrifuged at 1000 g for 20 min to remove debris. HGF, KGF and bFGF concentrations were quantified using the commercially available human growth factor ELISA kits (Cloud-Clone Crop, China), according to the manufacturer’s instructions. Absorbance was determined at 450 nm on a microplate reader (Tecan, Reading, UK) using Magellan software (Tecan, Reading, UK).

#### Extracellular matrix remodeling

hGFs were seeded onto different Ti samples in 24-well plates with 1.5 × 10^5^ cells/well and cultured for 4 and 24 h. Total RNA was extracted using the Trizol reagent (Takara, Japan). The purity and concentration of the total RNA were measured using the Nanodrop 2000 (Thermo Fisher Scientific, USA). After reverse transcription, quantitative Real-time Polymerase Chain Reaction (qRT-PCR) was conducted to detect the mRNA expression of metalloproteinases-1 (MMP-1), MMP-3 and tissue inhibitors of MMP-1 (TIMP-1) according to the manufacturer’s protocols (Accurate, China). The primers used in this study are presented in Table 1.

**Table 1.** The primers sequences for RT-qPCR analysis

Gene	Forward primer sequence (5′–3′)	Reverse primer sequence (3′–5′)
MMP-1	CACGCCAGATTTGCCAAGAG	CCCCTCTAGTAGCCCTGTTG
MMP-3	TGGACAAAGGATACAACAGGGAC	CCAAGGCGGACAGAGTTCTA
TIMP-1	ATTCCGACCTCGTCATCAGG	CGCCTATGAAGGTGTCCAGG

### Bacteria experiments

#### Bacteria culture

*Porphyromonas gingivalis (Pg)* strain was revived and inoculated onto Brain-Heart Infusion Agar (BHIA) medium containing 10% sheep blood, vitamin K and hemin. The medium was incubated anaerobically at 37°C for 7 days. A single colony was picked and streaked onto fresh solid medium, followed by incubation under the same conditions for another 7 days. An appropriate number of colonies were then suspended in Brain-Heart Infusion Broth (BHI) medium and incubated for 24 h. The bacteria suspension was then counted using a turbidimeter and diluted as needed.

*Streptococcus mutans (Sm)* strain was revived on BHIA medium and streaked. The medium was incubated at 37°C for 2 days. A single colony was picked and streaked onto fresh medium, followed by incubation under the same conditions for another 2 days. An appropriate number of colonies was then suspended in BHI medium and incubated for 24 h. The bacterial suspension was then counted using a turbidimeter and diluted as needed.

#### Bacteria morphology

The Ti samples were placed into 24-well plates. One milliliter of bacterial suspension (1 × 10^4^ CFU/mL) was added into each well and cultured under the appropriate condition for 24 h. The surface-adhered bacteria were fixed with 2.5% glutaraldehyde, dehydrated with gradient ethanol and incubated with hexamethyldisilazane for 30 min. The samples were then dried in the fume hood, and sputter-coated with gold, and observed under FE-SEM to observe the morphology of adhesive bacteria.

#### Bacteria viability

The Ti samples were placed into 24-well plates. One milliliter of bacteria suspension (1 × 10^6^ CFU/mL) was added into each well and cultured under the appropriate condition for 48 h. Then the culture medium was discarded, and the samples were washed with PBS three times. Five hundred microliters of live/dead bacterial staining reagent diluent (live bacteria were stained green with Syto9, while dead bacteria were stained red with propidium iodide) were added into each well and incubated in the dark for 15 min. The live/dead bacteria labeled with a fluorescent dye were observed under CLSM within 1 h. ImageJ software was used to analyze the fluorescence intensity.

### Animal experiments

#### Establishment of rat implant model

All animal experiments were approved by the Animal Ethics Committee of The Fourth Military Medical University (IACUC-20241486). Twenty-four Sprague–Dawley rats (male, 6 weeks, 150–200 g) were included and randomly distributed into four groups to construct the implant model after tooth extraction. The threaded pure Ti implants with an integrated abutment were provided by Baoji Titanium Industry Co., Ltd (3 mm in length, 2 mm in diameter). The implants with different surface characteristics were prepared and characterized as described in the section ‘Material preparation and characterization’, and also divided into four groups: P, NT, PD@NT and LL-37-PD@NT (Based on the results of the *in vitro* migration experiment, the loading concentration of LL-37 was chosen as 25 μg/mL for the *in vivo* experiment).

The rats were anesthetized by intraperitoneal injection of pentobarbital sodium. Then the left maxillary first molar was extracted, and the implant was screwed into the tooth socket with the abutment across the gingiva mucosa and partially exposed in the oral cavity. The rats were allowed to recover and resume normal diet for another 4 weeks.

#### Micro-computed tomography scan

To determine the suitability of the implantation site, we first sacrificed a healthy rat and obtained maxillary bone samples to examine the position of the maxillary first molar. Subsequently, another rat was sacrificed immediately after tooth extraction to evaluate the condition of the extraction socket. Finally, a third rat was sacrificed 4 weeks after implantation to assess the position and stability of the implant. The aforementioned samples were fixed using 4% PFA. Micro-computed tomography (micro-CT) scan was performed on the maxillary bone samples to observe the changes in the alveolar socket and confirm the successful construction of implantation model. The scan parameters were as follows: effective pixel size: 39.05 μm, exposure time: 1000 ms, current: 500 μA, voltage: 80 kV. The scanning data were reconstructed using Materialise’s interactive medical image control system.

#### Tissue sample harvesting and hematoxylin–eosin staining

The experimental rats were sacrificed 4 weeks post-implantation, with maxillary bone samples collected. Then the samples were fixed in 4% PFA for 24 h and decalcified in ethylenediaminetetraacetic acid solution at 37°C for 1 month. Then the implants were carefully removed to minimize the damage to the surrounding soft tissues. The tissue samples without implant were embedded in paraffin, and 5-μm-thick sections were prepared along the buccal-palatal direction using a microtome. The sections were deparaffinized in xylene and hydrated in gradient alcohol, followed by hematoxylin–eosin (HE) staining. The histological morphology and structure of peri-implant soft tissues were observed by an optical microscope.

#### Immunohistochemical staining

The sections were deparaffinized and hydrated as the section ‘Tissue sample harvesting and hematoxylin–eosin staining’. Antigen retrieval was performed in antigen repair buffer, followed by serum blocking. Rabbit-enhanced polymer detection system was used to detect type I collagen (Col I, ABclonal, China) in the sections. The histological images were observed and collected under an optical microscope, and ImageJ software was used for analyzing the intensity and range of positive expression of the interest protein.

#### Horseradish peroxidase infiltration experiments

To assess the closure strength of peri-implant soft tissues around the abutment, an horseradish peroxidase (HRP) infiltration experiment was designed [24]. Briefly, 1 h prior to sacrifice, the rats were administered general anesthetic via intraperitoneal injection of sodium pentobarbital. A cotton thread was soaked into 50 mg/mL HRP solution and then placed onto the gingiva mucosa around the abutment. For every 10 min, 10 μL HRP solution was dropped onto the cotton thread using a pipette. One hour later, the rats were sacrificed and maxillary bone samples were collected, and the tissue sections were prepared as described in the section ‘Tissue sample harvesting and hematoxylin–eosin staining’. After washing with PBS and staining with rabbit-enhanced polymer detection system, the infiltration range of HRP into peri-implant soft tissues was observed by an optical microscope and evaluated using Image J software.

### Statistical analysis

Data were expressed as mean±SD. One-way ANOVA was used to compare the overall differences, and LSD-*t* test was used for pairwise comparisons. The level of significance was set at bilateral α = 0.05.

## Results and discussion

### Preparation and characterization of LL-37-PD@NT

**Figure 3.**
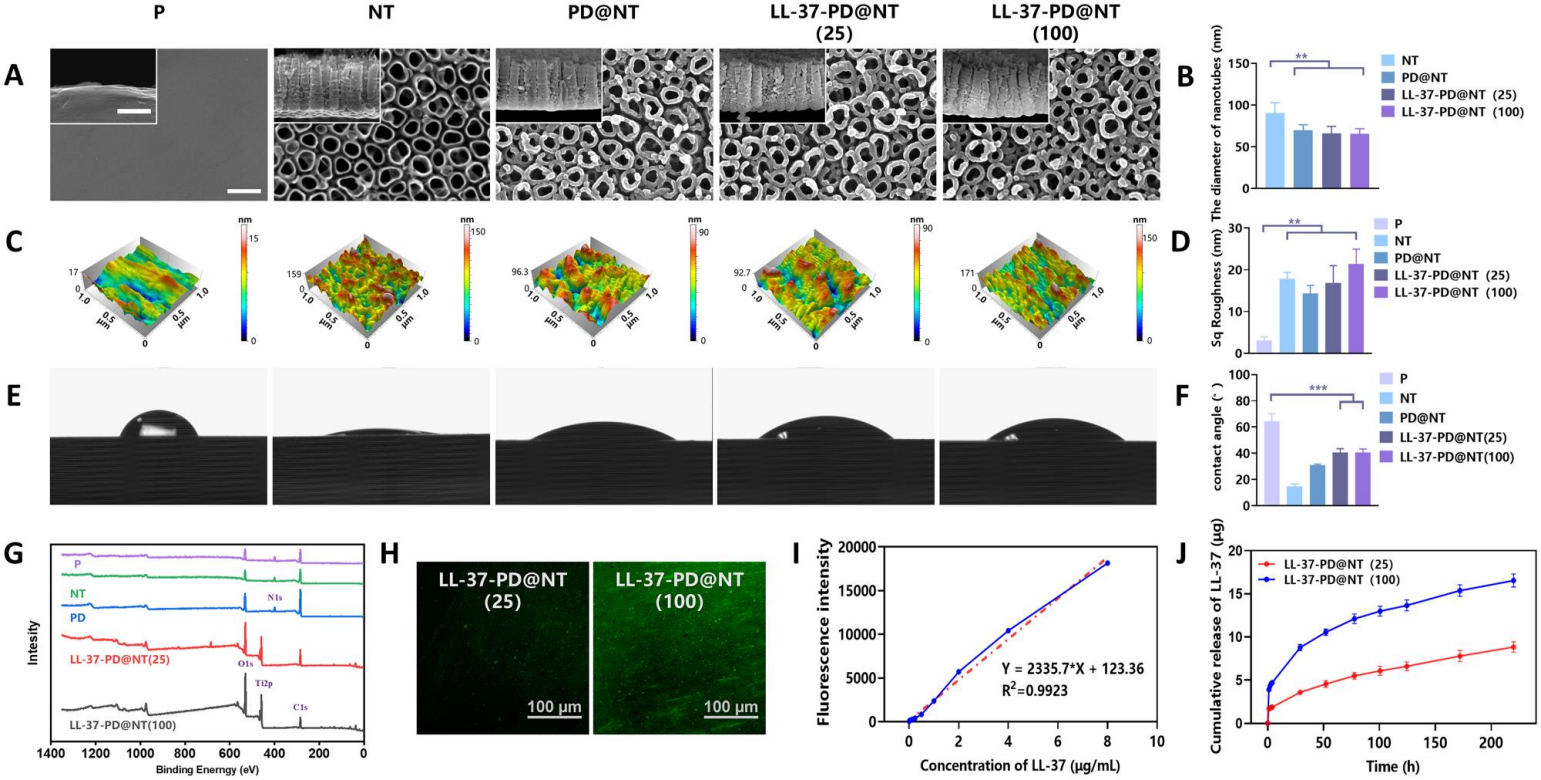
Characterizations of Ti samples. (**A**) Surface topography of different Ti samples was examined using FE-SEM. Bar: 400 and 200 nm. (**B**) Diameter analysis of nanotubes on the surface of different Ti samples. (**C**) Surface roughness of different Ti samples was examined using AFM. (**D**) Analysis of surface Sq roughness. (**E, F**) The hydrophilicity of different Ti samples was evaluated by the water contact angles. (**G**) The elemental composition of Ti samples was detected by XPS. (**H**) Load of FITC-labeled antimicrobial peptides was observed using fluorescence microscopy (**I**) Standard curve of antimicrobial peptide release. (**J**) *In vitro* release profile of antimicrobial peptide on the surface of different Ti samples. The data were presented as mean ± SD with a sample size of *N* = 3. Statistical significance was indicated as **P < 0.01 and ***P < 0.001.

In this study, electrochemical anodization was employed to fabricate nanotube arrays on the surface of P samples. As shown in Figure 3, the NT samples exhibited a regular arrangement of nanotubes with a diameter of approximately 100 nm. Subsequently, a significant reduction in nanotube diameter and an increase in tube wall thickness were observed on the PD@NT group after being coated with the PD layer. After further loading of LL-37 (LL-37-PD@NT), the surface morphology of Ti samples, including the diameter of nanotubes was almost unchanged (Figure 3A and B).

Simultaneously, AFM was used to measure the surface roughness of different Ti samples. The roughness was expressed in terms of root mean square height, and the value of P, NT, PD@NT, LL-37-PD@NT(25) and LL-37-PD@NT(100) was 3.02 ± 0.92 nm, 17.77 ± 1.65 nm, 14.30 ± 1.95 nm, 16.85 ± 4.17 nm and 21.35 ± 3.60 nm, respectively. The above results suggest that anodization treatment could significantly increase the roughness of Ti samples. Meanwhile, after being coated with PD, the roughness of Ti samples decreased, but the loading of LL-37 further enhanced the surface roughness of Ti samples. However, the variation in roughness among the different nanotube groups was minimal and showed no statistical significance (Figure 3C and D).

Furthermore, a contact angle goniometer was used to measure the wettability of different Ti samples. The contact angles of the five groups of Ti samples were 64.6° ± 5.6°, 14.7° ± 2.0°, 30.7° ± 1.1°, 40.6° ± 2.9° and 40.6° ± 2.7°, respectively, which suggests that anodization treatment could significantly increase Ti surface hydrophilicity compared with P group, while further PD and LL-37 coating slightly reduced Ti surface hydrophilicity, but is still significantly superior to P group (Figure 3E and F).

Finally, XPS was performed to analyze the surface chemical element composition of different Ti samples. The elements of Ti and O were dominant in P and NT groups. After being coated with PD, the Ti peak disappeared and the N peak appeared, suggesting that the nanotube substrate was completely covered. After LL-37 loading, the C and N peaks were enhanced, which was attributed to the amino groups of LL-37 molecule. The results further indicated that the PD and LL-37 were successfully immobilized onto the surface of nanotubes (Figure 3G). PD coating is widely used for protein immobilization, yet its interaction mechanism remains debated [21]. On one hand, PD can immobilize biomolecules through non-covalent interactions such as hydrogen bonding, electrostatic forces and affinity adsorption, thereby enabling secondary surface functionalization [25, 26]. On the other hand, owing to its abundant catechol and quinone functional groups, PD is capable of covalently grafting functional molecules via Michael addition or Schiff base reactions with thiol (-SH) or amino (-NH2) groups [27, 28]. In this study, we hypothesize that the amino groups in peptide LL-37 form covalent bonds with quinones in the PD coating through Michael addition or Schiff base reactions, although electrostatic or other non-covalent interactions between the peptide and PD cannot be entirely ruled out. Nevertheless, the exact binding modes between LL-37 and the PD interface still require further investigation.

LL-37 labeled with FITC was employed to observe the loading and releasing characteristics of peptide. The observation under CLSM revealed that scattered green fluorescent spots with different sizes could be found on the surfaces of LL-37-PD@NT (25) and LL-37-PD@NT (100) (Figure 3H). The fluorescence intensity and spot density in the LL-37-PD@NT (100) group were enhanced compared to those in the LL-37-PD@NT (25) group. The *in vitro* cumulative release curve showed an initial burst release (1–24 h) followed by a sustained release process of peptide over a long term (2–8 days) (Figure 3J). After 8 days, the release of peptide could still be detected. The cumulative release amounts of peptide from the LL-37-PD@NT (25) and LL-37-PD@NT (100) groups reached 3.6 and 8.8 μg at 24 h, respectively (Figure 3I).

### Cell experiments

#### Effects of LL-37-PD@NT on the chemotaxis and early adhesion of hGFs

**Figure 4.**
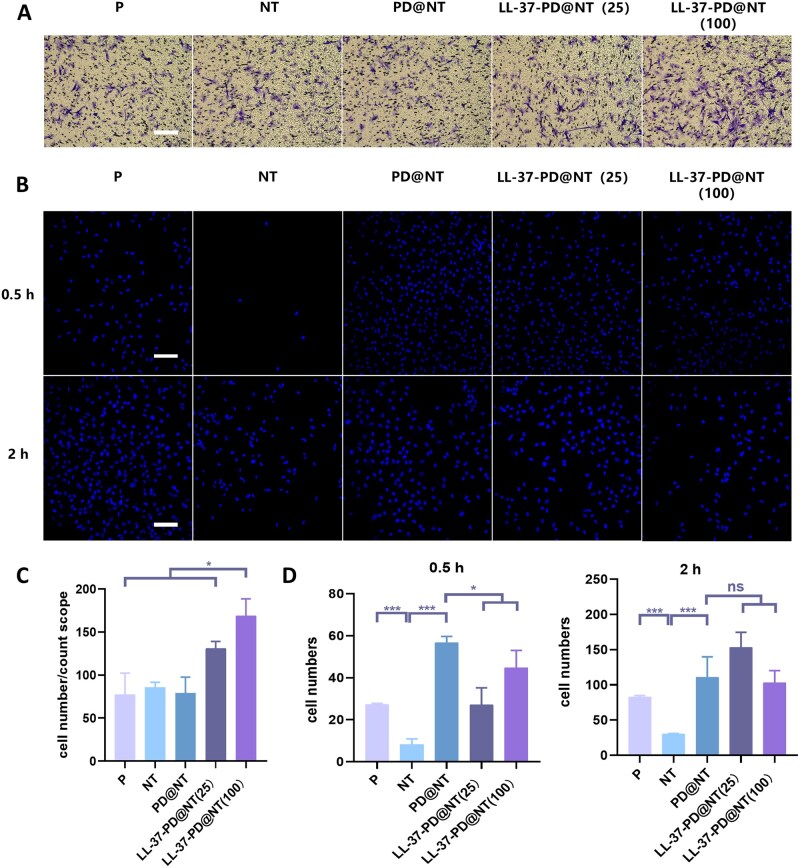
The effect of different Ti samples on the chemotaxis and early adhesion of hGFs. (**A, C**) Observation and quantitative analysis of hGFs migrated to the lower chamber of Transwell after 12 h. Bar: 200 μm. (**B, D**) Observation and quantitative analysis of hGFs adhered onto the surface of different Ti samples after incubation for 0.5 and 2 h (blue fluorescence indicates the cell nucleus). Bar: 100 μm. The data were presented as mean ± SD with a sample size of *N* = 6. Statistical significance was indicated as *P < 0.05, ***P < 0.001 and ns means no significance.

The vertical migration assay mediated by Transwell chambers was performed to investigate the chemotactic ability of LL-37-PD@NT to hGFs **(**Figure 4A). The results suggested that the amount of hGFs migrated to the lower chamber in P, NT and PD@NT groups had little difference, while LL-37-PD@NT (25) and LL-37-PD@NT (100) could induce a larger amount of hGFs migrating, especially the latter group, which was significantly more than other groups (Figure 4C). The loading concentration of LL-37 affected its ability of chemotactic cells. Additionally, other studies have discovered that the effective chemotactic concentration of LL-37 is 0.1–11 mM, and its chemotactic effect is highly correlated with the local concentration, which is consistent with this study [16].

Immunofluorescence staining was used to observe the early adhesion of hGFs on Ti samples. At 0.5 and 2 h, the number of adherent cells in NT group was significantly reduced compared to P group (*P* < 0.01). Meanwhile, the PD@NT group had an increased number of adherent cells than that of P group (*P* < 0.001). Compared with PD@NT group, less-adherent cells were observed on LL-37-PD@NT (25) and LL-37-PD@NT (100) groups at 0.5 h (*P* < 0.05). However, there was no statistically significant difference in adherent cells among PD@NT, LL-37-PD@NT (25) and LL-37-PD@NT (100) groups at 2 h, yet LL-37-PD@NT (25) group showed a trend of more adherent cells (Figure 4B and D). After implantation, various components of plasma proteins would be absorbed onto the material surface to form a structure similar to the extracellular matrix (ECM), thus promoting host cell adhesion [29]. The PD coating can adsorb proteins through covalent or non-covalent interactions [21, 25, 27]. In this study, LL-37-PD@NT significantly promoted the early adhesion of hGFs, which might be related to the strong fixation effect of PD on ECM proteins [30].

hGFs are the primary cellular component of the connective tissue attachment around the implant abutment. As documented, hGFs migrate to the wound site after implantation and secrete ECM matrix through various signaling pathways, and participate in the healing and remodeling of peri-implant gingival tissues [31, 32]. Consequently, hGFs play a crucial role in the formation of soft tissue seal around implants. Due to the lack of hGFs in the peri-implant tissue compared to natural teeth, improving the adhesion of hGFs through surface modification of implant materials has been the focus of many studies [12, 33]. In this study, the results indicated that the loading of LL-37 exhibited a stronger chemotactic effect on hGFs. Furthermore, 2 h after cell seeding, the LL-37-PD@NT (25) group showed a trend toward increased number of adherent cells. To investigate the potential mechanisms underlying this adhesion phenomenon, qRT-PCR analysis was employed to assess the expression levels of adhesion-related genes-fibronectin (FN). Detailed experimental information is described in the Supplementary Section S1.2. Although no significant upregulation of FN was detected in hGFs cultured on LL-37-biofunctionalized Ti samples relative to the P samples (Supplementary Figure S2), our findings indicate that the modified surface promotes both the recruitment and adhesion of hGFs, which could be beneficial for subsequent formation of soft tissue seal.

#### Effects of LL-37-PD@NT on the morphology and proliferation of hGFs

**Figure 5.**
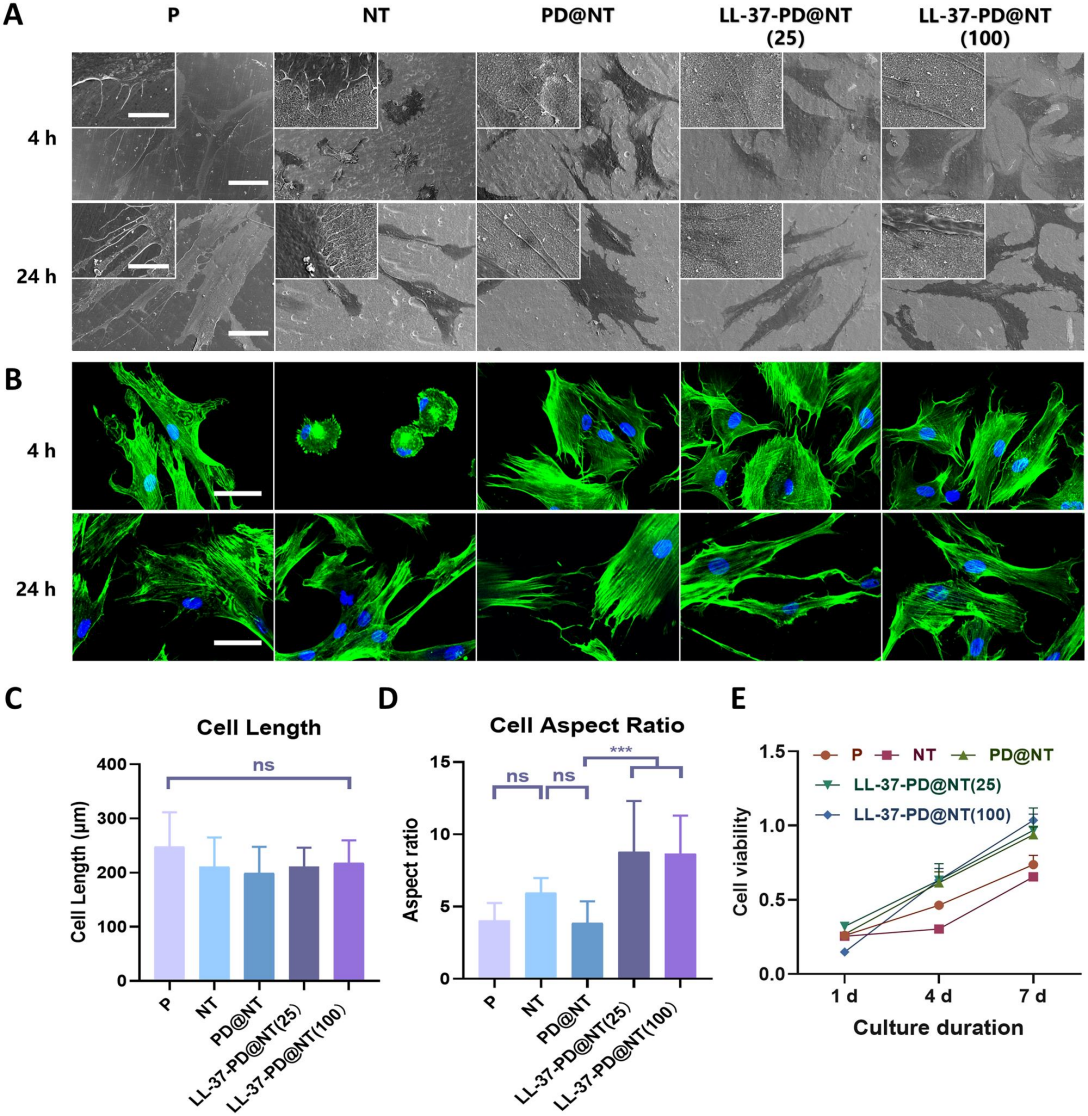
The effect of different Ti samples on the morphology, cytoskeleton extension and proliferation of hGFs. (**A**) FE-SEM observation of hGFs on the surface of different Ti samples after incubation for 4 and 24 h. Bar: 5 and 50 μm, respectively. (**B**) Cellular immunofluorescence observation of the cytoskeleton extension of hGFs on the surface of different Ti samples after incubation for 4 and 24 h (blue fluorescence indicates the cell nucleus, green fluorescence indicates the actin filaments). Bar: 50 μm. (**C, D**) Analysis of cell length and aspect ratio of hGFs on the surface of different Ti samples after incubation for 24 h. (**E**) Cell viability analysis of hGFs on the surface of different Ti samples after incubation for 1, 4 and 7 days. The data were presented as mean ± SD with a sample size of *N* = 6. Statistical significance was indicated as ***P < 0.001 and ns means no significance.

In addition to the number change of adherent cells, hGFs’ morphology and biological behavior were also changed once they were adhered to the surface of materials [34]. hGFs were seeded on different Ti samples and cultured. After 4 h, FE-SEM images indicated that the cells exhibited large spread area and spread out in a spindle shape with long filopodia, except for the cells in NT group that were irregular or round with a small spread area and short filopodia. However, cells of all groups were fully spread at 24 h (Figure 5A). The immunofluorescence staining results were similar to those observed under FE-SEM. At 24 h, hGFs on the surface of different Ti samples appeared elongated (Figure 5B). Using ImageJ software, we conducted semi-quantitative analysis of the length and aspect ratio of cells. There was no statistically significant difference of cell length among different groups (Figure 5C), but the aspect ratio of cells in LL-37-PD@NT (25) and LL-37-PD@NT (100) groups was significantly increased, indicating a trend towards elongation at both ends (Figure 5D). Research indicates that the cellular functions of fibroblasts on the surface of materials are related to their elongation rate, and when the elongation rate is within a certain range, the cellular functions are the most vigorous [35]. In this study, hGFs on LL-37-PD@NT exhibited the most obvious trend of bipolar elongation, suggesting that the cell functions had been significantly improved.

As MTT assay results showed, the proliferation ability of hGFs in NT group reduced compared to P group at 4 and 7 days; however, the PD coating and LL-37 loading could significantly improve cell viability. Furthermore, there was no statistically significant difference of cell proliferation in PD@NT, LL-37-PD@NT (25) and LL-37-PD@NT (100) groups, suggesting that PD-mediated LL-37 loading could not influence the viability of hGFs (Figure 5E).

#### Effects of LL-37-PD@NT on the migration, growth factor secretion and ECM remodeling of hGFs

**Figure 6.**
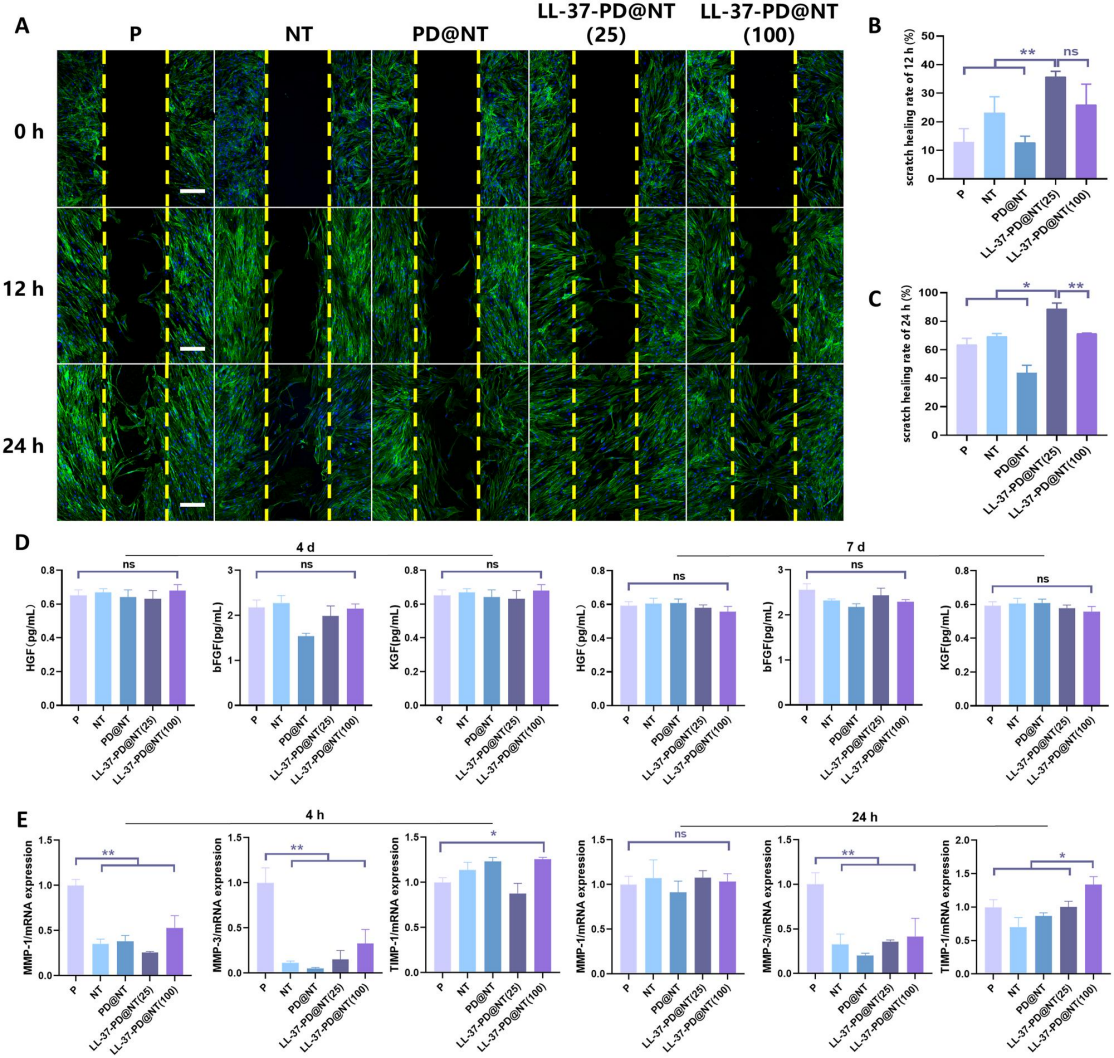
The effect of different Ti samples on the migration, growth factor secretion and ECM remodeling of hGFs. (**A**) The wound scratch assay was used to detect the migration of hGFs on the surface of different Ti samples after culturing for 0, 12 and 24 h (blue fluorescence indicates the cell nucleus; green fluorescence indicates the actin filaments). Bar: 200 μm. (**B, C**) Statistical analysis of the scratch healing rates at 12 and 24 h. (**D**) ELISA was used to measure the secretion of HGF, bFGF and KGF from hGFs on the surface of different Ti samples after incubation for 4 and 7 days. (**E**) qRT-PCR analysis of mRNA expressions of MMP-1, MMP-3 and TIMP-1 of hGFs on the surface of different Ti samples after incubation for 4 and 24 h. The data were presented as mean ± SD with a sample size of *N* = 3. Statistical significance was indicated as *P < 0.05, **P < 0.01 and ns means no significance.

The wound scratch results manifested that cells at scratch edge began to migrate towards the center at 12 h and the cells in LL-37-loaded groups showed strong migration ability (Figure 6A and B). Within 24 h, hGFs in LL-37-PD@NT (25) group had the fastest migration rate among the five groups (Figure 6C). At 24 h, the scratch had been filled with monolayer cells in LL-37-PD@NT (25) group (Figure 6A). Nevertheless, this optimal concentration differed from the results of the chemotaxis experiments. The difference in results might be related to different experimental methods and the difference in the concentration of LL-37 that hGFs actually perceive. Specifically speaking, hGFs attached to the upper chamber and had a certain distance from the lower materials in the Transwell, thus the diffusion gradient of antimicrobial peptides in the solution enabled hGFs to sense different concentrations of LL-37, which might be different from the concentration of LL-37 perceived by hGFs in the scratch assays. However, it is unquestionable that the LL-37-PD@NT did have strong chemotaxis to hGFs and could promote hGFs’ migration on the surface of Ti samples, thus accelerating the formation of soft tissue seal.

Following implantation, as a part of the healing process, fibroblasts can be recruited to the injured site and transformed into myofibroblasts upon activation, synthesizing and secreting ECM and various growth factors [36]. After the initial deposition of ECM, fibroblasts can remodel ECM by secreting some enzymes, such as matrix metalloproteinases (MMPs) [37, 38]. In this study, qRT-PCR results revealed that the mRNA expression of MMP-3 and MMP-1 of hGFs in LL-37-PD@NT was decreased, while the early expression of tissue inhibitors of TIMP-1 was increased, which was conducive to ECM synthesis and remodeling and beneficial to enhance the strength of soft tissue healing (Figure 6E). HGF, bFGF and KGF play critical roles in the healing and remodeling processes of periodontal tissues [17, 39]. However, ELISA results verified that there was no significant difference in the secretion of HGF, bFGF and KGF from hGFs in different groups either in 4 days or in 7 days, suggesting that LL-37 may not affect the growth factor secretion of hGFs (Figure 6D). In this case, the future functionalization of LL-37@PD-NT may need to be restricted to specific application scenarios. For instance, in pathological conditions where hGFs’ functions are impaired, such as in diabetes [40], the limited secretion of cytokines may not yield satisfactory results.

### Bacteria experiments

**Figure 7.**
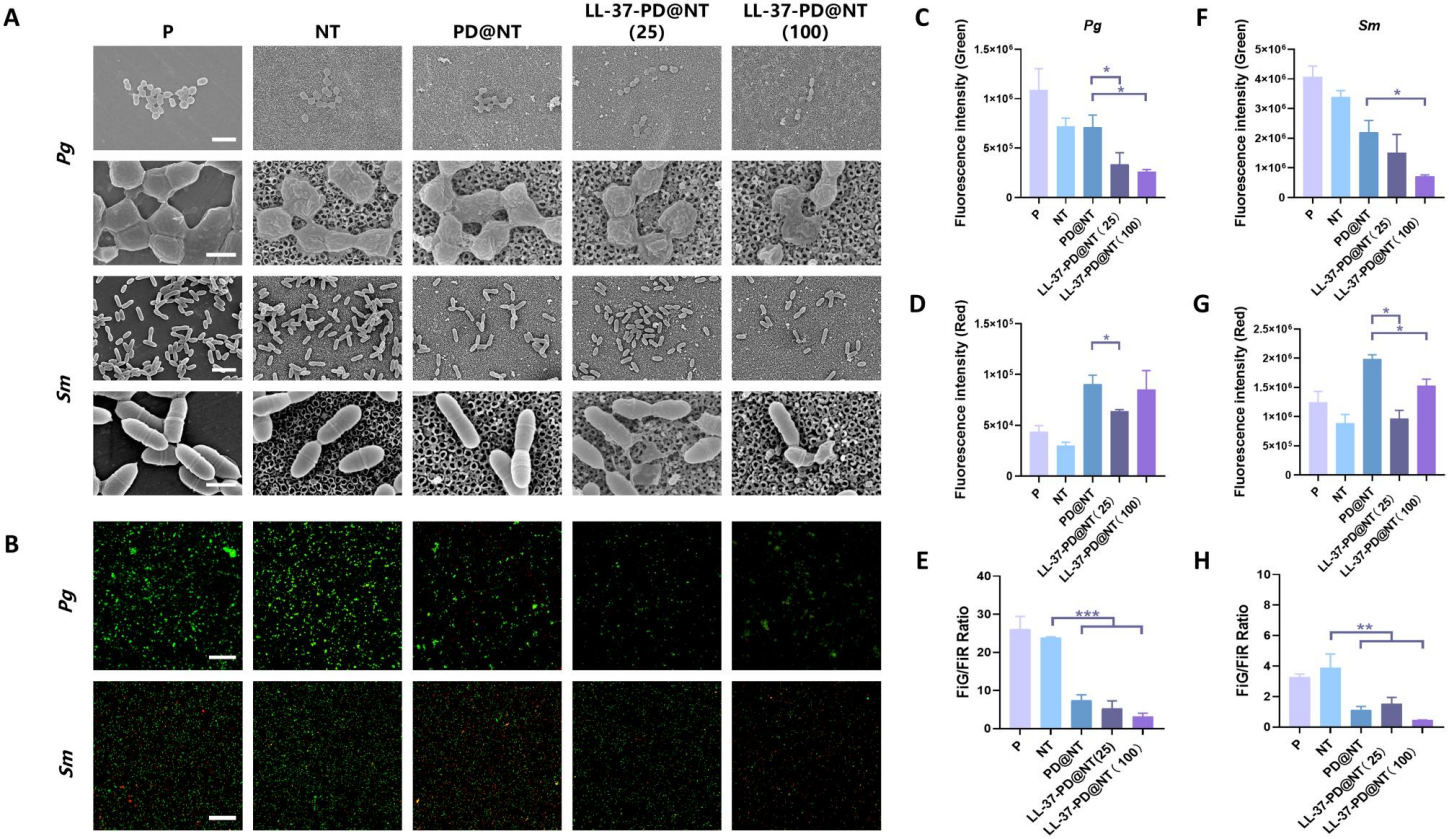
Evaluation of antibacterial properties of different Ti samples. (**A**) FE-SEM observation of *Pg* and *Sm* on the surface of different Ti samples after incubation for 24 h. Bar: 2 μm and 500 nm. (**B**) Distribution of the live/dead *Pg* and *Sm* on the surface of different Ti samples (green for live cells, red for dead cells. Bar: 100 μm). Semi-quantitative analysis of the fluorescence intensity and the ratio of live/dead *Pg* (**C, D, E**) and *Sm* (**F, G, H**) on the surface of different Ti samples. The data were presented as mean ± SD with a sample size of *N* = 3. Statistical significance was indicated as *P < 0.05, **P < 0.01 and ***P < 0.001.

After the implant was placed in the mouth, it will be exposed to a bacterial environment in the oral cavity. In the research of antibacterial properties, *Pg* (a common pathogen associated with periodontitis) and *Sm* (a common oral bacterium) were selected to investigate the antibacterial properties of LL-37-PD@NT. LL-37 is a typical cationic antimicrobial peptide with strong inhibitory effect on both Gram-negative and Gram-positive bacteria [18]. FE-SEM were used to observe the morphology of bacteria on the surface of different Ti samples. The results showed that *Pg* and *Sm* on the surface of P group appeared in good condition with a complete cellular structure and tight interbacterial connections. However, once *Pg* and *Sm* were inoculated on the surface of modified Ti samples, the bacterial morphology gradually changed with shrinkage and sparse interbacterial connections. Particularly, the bacteria in LL-37-PD@NT (25) and LL-37-PD@NT (100) groups exhibited significant shrinkage, even with bacterial rupture (Figure 7A), which indicated that LL-37 still maintained good antibacterial performance after it was fixed on the Ti surface by the dopamine intermediate coating method.

The live/dead bacterial staining revealed that the green fluorescence intensity became weaker along with the surface modification of Ti samples (Figure 7B). The number of viable bacteria adhering to the Ti samples in LL-37-PD@NT groups decreased significantly. Meanwhile, compared with P and NT groups, the ratio of green to red fluorescence intensity in PD@NT, LL-37-PD@NT (25) and LL-37-PD@NT (100) groups significantly decreased, especially in the LL-37-PD@NT (100) group (Figure 7C–H), thereby indicating its superior antibacterial efficacy.

Regarding the mechanisms of antimicrobial peptides, it is widely accepted that they not only exert direct bactericidal effects by targeting bacterial cell walls, cell membranes, nucleic acids and proteins but also participate in immune regulation to achieve indirect antibacterial actions [19]. In this study, FE-SEM observations revealed significant morphological alterations in bacteria adherent to the Ti samples coated with LL-37-PD@NT (25) and LL-37-PD@NT (100), including sparse interbacterial connections, shrinkage and even rupture of bacterial body. These findings suggest that the antimicrobial peptide LL-37 exerted direct antibacterial effects by disrupting the cell wall and membrane integrity of bacteria adhered to the samples, ultimately leading to bacterial death. The live/dead bacterial staining revealed that the number of viable bacteria adhering to the Ti samples in LL-37-PD@NT group decreased significantly. Considering the early burst release and long-term sustained release effects of LL-37 found in material characterization tests, we supposed that LL-37 released into the surrounding environment also exerted an antibacterial effect, thus inhibiting re-colonization of bacteria [41].

### Animal experiments

**Figure 8.**
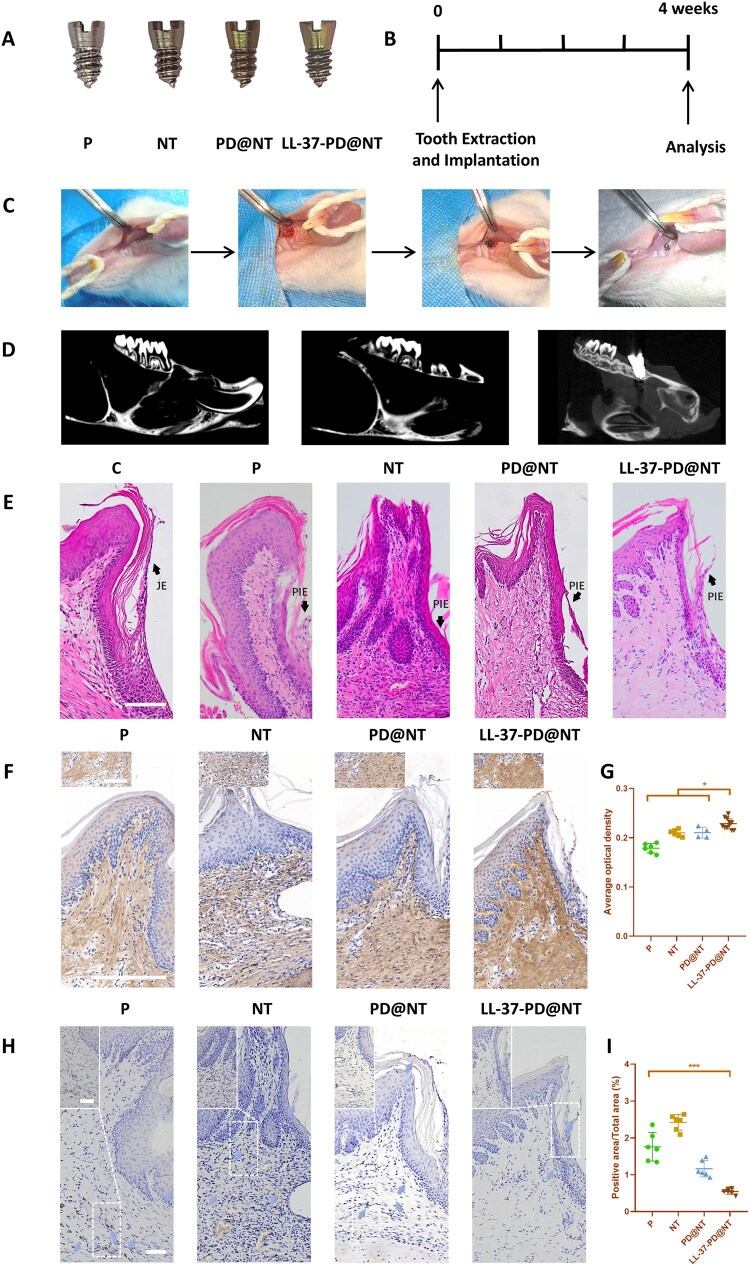
The effect of different Ti samples on the peri-implant soft tissue seal of rats. (**A**) Photographs of different Ti implants. (**B**) Timeline for the *in vivo* studies. (**C**) Surgical procedures of tooth extraction and implantation. (**D**) Micro-CT scanning observation of the maxillary first molar of rats before and after extraction, and 4 weeks after implantation. (**E**) Histological observation of the peri-implant soft tissues by HE staining (the control image showing the native teeth and the arrows indicate the junctional epithelium of the natural tooth and the PIE, respectively). Bar: 100 μm. (**F**) Histological observation of col I expression in the peri-implant tissues by immunohistochemistry staining. Bar: 100 μm. (**G**) Semi-quantitative analysis of the immunohistochemistry staining results. (**H**) The strength of peri-implant soft tissue seal was measured by HRP infiltration experiments. Bar: 100 μm. (**I**) Semi-quantitative analysis of the HRP experiments. The data were presented as mean ± SD with a sample size of *N* = 6. Statistical significance was indicated as *P < 0.05 and ***P < 0.001.

Micro-CT analysis showed that the bone wall of alveolar socket remained intact after extraction of the maxillary first molar. Four weeks after implantation, the implant was still in place and the osseointegration had been formed, with the abutment across the gingiva (Figure 8D). HE staining displayed that the peri-implant epithelium (PIE) had formed around the abutment in all groups. Typically, the composition and function of the epithelial and connective tissue attachments around implants were significantly different from those of natural teeth [42–44]. However, the junctional epithelium formed along the LL-37-PD@NT group after 4 weeks of implantation was similar to the junctional epithelium found in natural teeth (Figure 8E).

Furthermore, the expression of collagen fibrils in the peri-implant connective tissues is detected by immunohistochemical staining. Compared with the P, NT and PD@NT groups, the positive expression rate of Col I in LL-37-PD@NT group was significantly increased, indicating that LL-37-PD@NT could induce the rat gingiva fibroblasts to secrete more collagen fibers (Figure 8F and G). The HRP infiltration assay was used to evaluate the closure strength of peri-implant soft tissues [24, 45]. The staining results showed that the brown deposits generated by HRP reaction were observed extensively both in the PIE and in the peri-implant connective tissues of P, NT and PD@NT groups; in contrast, in the LL-37-PD@NT group, almost all the brown deposits were limited to the PIE, and only a small amount of brown deposits were found in the peri-implant connective tissues (Figure 8H). The semi-quantitative analysis showed that the area proportion of brown deposits in LL-37-PD@NT group was the lowest (Figure 8I), indicating its optimum sealing efficiency of peri-implant soft tissues. These results reinforced that LL-37-PD@NT could significantly enhance the early peri-implant soft tissue healing. In subsequent studies, we will extend the follow-up period to comprehensively access the long-term performance of LL-37—biofunctionalized Ti in promoting peri-implant soft tissue heal and improving success rate of implant.

## Conclusion

In this study, LL-37-biofunctionalized Ti based on PD intermediate coating method was constructed, and we confirmed that the new implant abutment material not only had good bactericidal and bacteriostatic properties, but also promoted the chemotaxis, adhesion, proliferation and migration of gingival fibroblasts, and even exerted a positive effect on the synthesis of ECM, which provided beneficial assistance for the competition between cells and bacteria on the surface of Ti materials. Most importantly, when LL-37-PD@NT was applied to the rat implantation model, we obtained a highly biomimetic and tight soft tissue seal. This research provides a promising application direction for the optimal design of implant abutment materials. Moreover, the comparatively simple fabrication process facilitates large-scale manufacturing and holds promise for clinical translation, representing a key focus of our ongoing research. However, significant challenges remain. For instance, the oral environment is rich in saliva, which contains a variety of proteases that may potentially compromise the bioactivity of peptide LL-37, and may even result in its inactivation. These effects are currently difficult to predict. Therefore, substantial obstacles must be overcome before clinical translation.

## Supplementary Material

rbaf117_Supplementary_Data
