# Supplementary material for: LL-37—biofunctionalized titanium improves soft tissue seal surrounding the dental implant from the perspective of optimizing a race to the surface
Source: Regen Biomater. 2025 Nov 12;12:rbaf117. doi: 10.1093/rb/rbaf117 (PMC12679594; doi:10.1093/rb/rbaf117)
Supplement: rbaf117_Supplementary_Data [file rbaf117_supplementary_data.zip › Supplementary-materials.docx]

**Supplementary materials**

LL-37 - biofunctionalized titanium improves soft tissue seal surrounding the dental implant from the perspective of optimizing a race to the surface

Yi Li^1a^, Junling Huang^2a^, Yan Zhang^1^, Yide He^1^, Dongxuan Cai^1^, Min Xu^1^, Qianli Ma^3^, Yumei Zhang*^1^ , Jinjin Wang*^2^

^1^ State Key Laboratory of Oral & Maxillofacial Reconstruction and Regeneration, National Clinical Research Center for Oral Diseases, Shaanxi Key Laboratory of Stomatology, Department of Prosthodontics, School of Stomatology, The Fourth Military Medical University, Xi’an, China

^2^ State Key Laboratory of Oral & Maxillofacial Reconstruction and Regeneration, National Clinical Research Center for Oral Diseases, Shaanxi Key Laboratory of Stomatology, Department of Periodontology, School of Stomatology, The Fourth Military Medical University, Xi’an, China

^3^Department of Biomaterials, Institute of Clinical Dentistry, University of Oslo, Oslo, Norway

1. **Materials and methods**

**1.1. Cell migration**

A scratch assay was employed to investigate the effect of LL-37 on human gingival fibroblasts (hGFs) migration. hGFs were seeded in 6-well cell culture plates with 3×10^5^ cells/well and cultured until a confluent cell layer formed. Then a scratch was generated in the cell monolayer using a 200 μL sterile pipette tip. The dislodged cells were gently washed away with PBS, and the medium was replaced with fresh culture medium containing different concentrations of LL-37 (0, 1.25, 2.5, 5 and 10 μg/mL). The scratch closure was observed and photographed under an optical microscope at 24 h and 48 h. The unhealing areas were quantified using the ImageJ wound-healing measurement tool. The healing rate was calculated by dividing the healing area by the total wound area.

**1.2. Real-time Polymerase Chain Reaction (qRT-PCR)**

hGFs were seeded onto different Ti samples in 24-well plates with 1.5×10^5^ cells/well and cultured for 24 h. Total RNA was extracted using the Trizol reagent (Takara, Japan). The purity and concentration of the total RNA were measured using the Nanodrop 2000 (Thermo Fisher Scientific, USA). After reverse transcription, quantitative Real-time Polymerase Chain Reaction (qRT-PCR) was conducted to detect the mRNA expression of fibronectin (FN) according to the manufacturer’s protocols (Accurate, China). The primers used in this study are presented in **Table S1**.

**Table S1.** The primers sequences for RT-qPCR analysis.

| Gene | Forward primer sequence (5’-3’) | Reverse primer sequence (3’-5’) |
| --- | --- | --- |
| FN | GAGGGCAGAAGAGACAACATGAA | CCCTTCATTGGTTGTGCAGATTT |


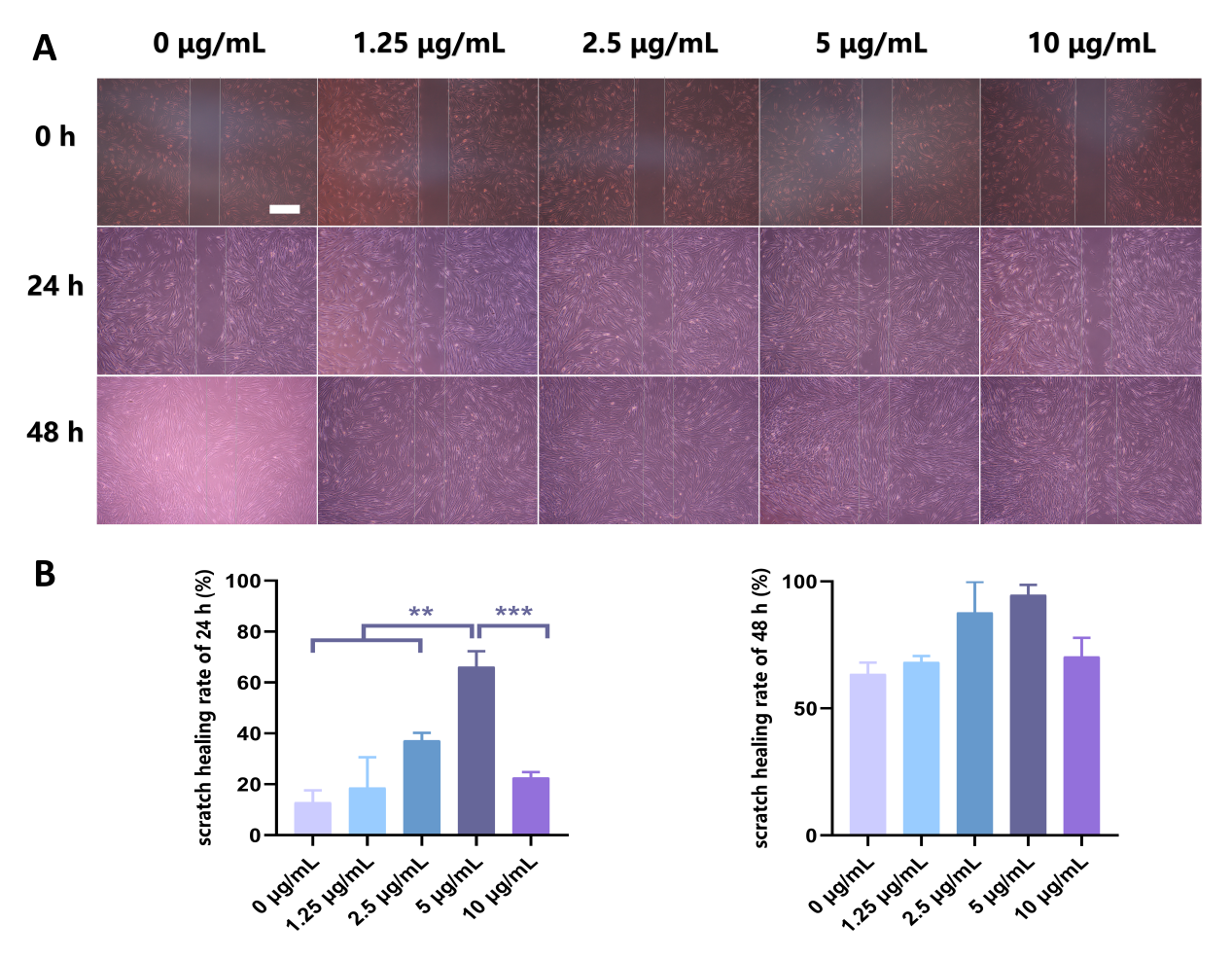


**Figure S1. The effect of LL-37 on hGFs migration.** (A) The wound scratch assay was used to detect the migration of hGFs after treatment with different concentrations of LL-37 at 0 h, 24 h, and 48 h. Bar: 500 μm (B) Statistical analysis of the scratch healing rates at 24 h and 48 h. The data were presented as mean ± SD with a sample size of N = 3. Statistical significance was indicated as ***p* < 0.01 and ****p* < 0.001.


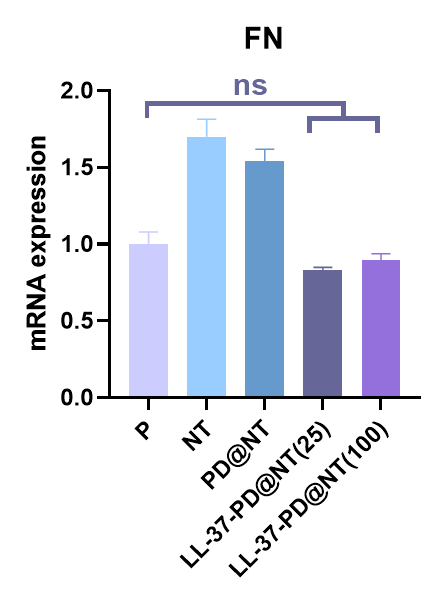


**Figure S2. qRT-PCR analysis of mRNA expression of FN of hGFs on the surface of different Ti samples after incubation for 24 h.** The data were presented as mean ± SD with a sample size of N = 3. Statistical significance was indicated as ns means no significance.
