# Supplementary figures and images for: LL-37—biofunctionalized titanium improves soft tissue seal surrounding the dental implant from the perspective of optimizing a race to the surface
Source: Regen Biomater. 2025 Nov 12;12:rbaf117. doi: 10.1093/rb/rbaf117 (PMC12679594; doi:10.1093/rb/rbaf117)

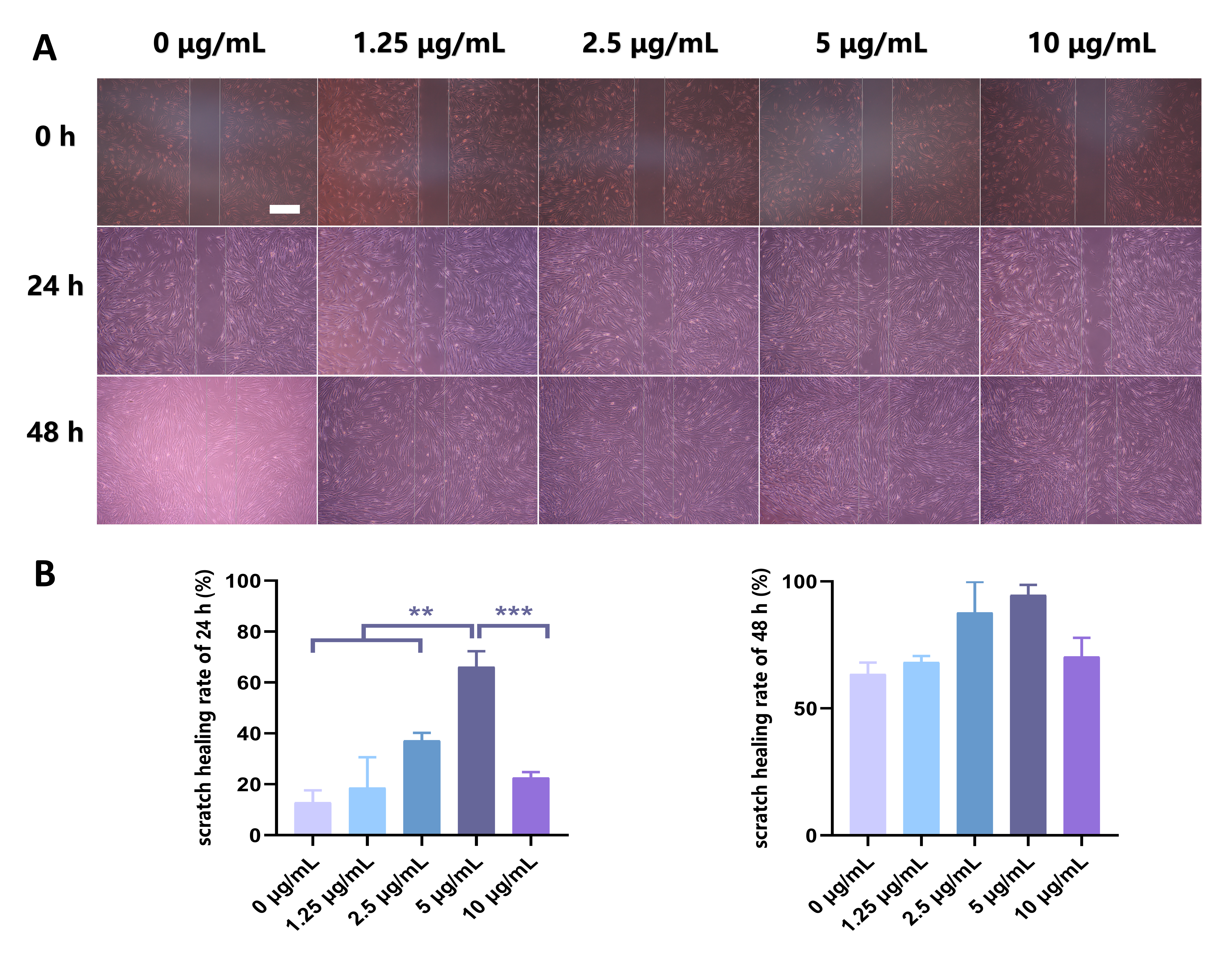

Supplement: rbaf117_Supplementary_Data [file rbaf117_supplementary_data.zip › FigureS1.tif]

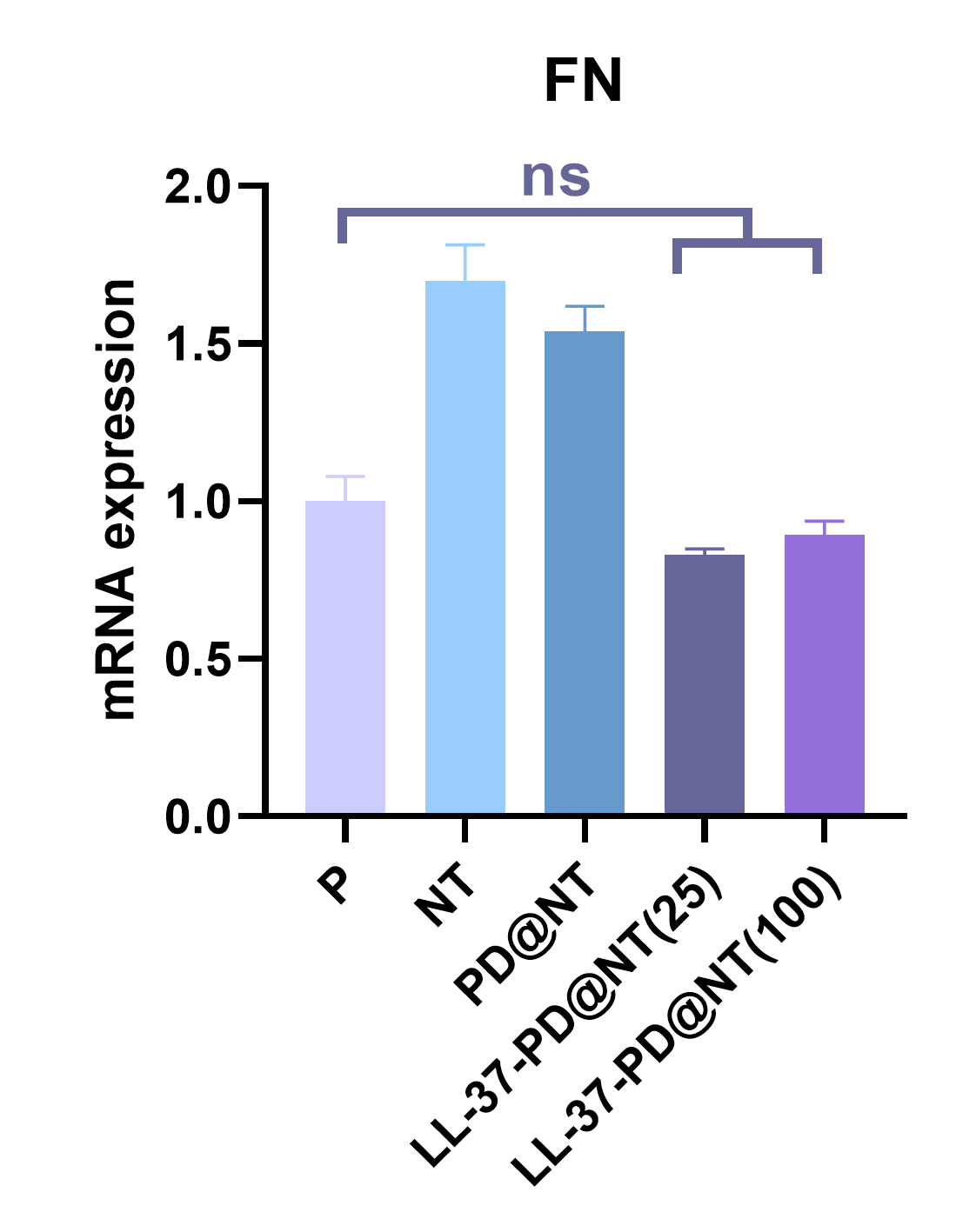

Supplement: rbaf117_Supplementary_Data [file rbaf117_supplementary_data.zip › FigureS2.tif]
